# *In vitro* comparison of the radiopacity of cavity lining materials with human dental structures

**DOI:** 10.4103/0972-0707.66713

**Published:** 2010

**Authors:** Fernanda CP Pires de Souza, Luiz C Pardini, Diogo R Cruvinel, Hisham M Hamida, Lucas FR Garcia

**Affiliations:** Department of Dental Materials and Prosthodontics, Ribeirao Preto School of Dentistry, University of São Paulo, Ribeirao Preto, SP, Brazil; 1 Department of Morphology, Stomatology, and Physiology, Ribeirao Preto School of Dentistry, University of São Paulo, Ribeirao Preto, SP, Brazil

**Keywords:** Calcium hydroxide, dentin, enamel, glass ionomer cement, radiopacity

## Abstract

**Aim:**

To compare the optical densities (OD) of calcium hydroxide (CH) and glass ionomer cement with the same thicknesses of the dental structures.

**Materials and Methods:**

Eighteen specimens of each material, with thicknesses of 0.5, 1.0, 1.5, 2.0, 2.5, and 3.0 mm were made in a Teflon matrix. To compare the radiopacity of the materials with the dental structures, dental cuts of the first molars, increasing in thickness from 0.5 to 3.0 mm, were obtained. To standardize the radiographs, a transparent acrylic matrix (Standardizing Device) was developed and used. Thirty radiographs were taken, five for each tested material.

**Results:**

Statistical analysis (Two-way ANOVA - Bonferroni, *P* < 0.05) demonstrated that when the materials were compared, there was statistically significant difference between the ODs, only for the thickness of 1.0 mm (*P* < 0.05).

**Conclusion:**

The thickness of the material contributed to its radiopacity, and these materials had to be used in a thickness between 1.5 and 2.0 mm.

## INTRODUCTION

Ideally, the materials used for dentin-pulp complex protection should present, among other properties, sufficient radiopacity, to be distinguished from the adjacent anatomical structures, such as, dentin and enamel.[1,2]

At present two materials are widely disseminated and used for this purpose: glass ionomer cements (GIC) and calcium hydroxide (CH); the latter has the properties of stimulating the formation of sclerotic and reparative dentin[1] and protects the pulp against thermal electric stimuli[2] and antibacterial action.[3,4] GIC also has several favorable properties and among them, the fluoride release that diminishes the incidence of secondary caries lesions, coefficient of thermal expansion similar to that of the tooth, pulp biocompatibility, and reduction in microleakage are pointed out.[5–7]

Ideally, the materials must present sufficient radiopacity to enable them to be differentiated from the adjacent anatomic structures,[8,9] facilitating the evaluation of restorations and enabling the detection of secondary caries,[10–15] because radiolucent materials may conceal the early diagnosis of some lesions.[11]

Several factors may affect the radiopacity of dental materials, but the composition seems to be the most important factor.[16] The higher the atomic number (Z) of the metal chemical elements present in the composition of the materials, higher is their capacity to absorb X-rays.[17] In addition, the material thickness,[13,14] angulation of the X-ray beam, methodology used for evaluation,[15] type of X-ray film, and radiographic processing can also have an influence.[18]

The degree of radiopacity required for an ideal clinical performance may vary according to the class of the material.[14] Some authors consider that the radiopacity of the material must be equal or higher than the radiopacity of dentin, to guarantee that the material is not confounded with carious dentin.[19] Others consider that the restorative material needs a slightly higher degree of radiopacity than enamel.[13–16,18] Nevertheless, several studies have assessed radiopacity from a 2 mm-increment graduated aluminum step-wedge, varying from 2 to 16 mm in thickness.[20–23]

The GIC is radiolucent because it is composed of light elements.[6,24] To increase its visibility in this sense, barium or zinc oxide glass is incorporated into it. In some products, the calcium (Z = 20) present in the composition has been replaced by strontium, which has a chemically similar behavior (Z = 38), and gives the material a greater capacity to absorb X-rays.[25]

However, CH cements are presented in the form of two components that set when they are mixed. The accelerator paste is composed of calcium hydroxide, zinc oxide, zinc stearate, and a vehicle. The base paste is composed of glycolic salicylate — the basic ingredient required for the setting reaction to occur — in addition to titanium dioxide, calcium sulfate, and calcium tungsten, which are inert particles serving as radiopacifiers.[26]

The aim of this study is to compare the optical densities of different thicknesses of pulp protecting materials (calcium hydroxide and glass ionomer cements) having the same thickness of dental structures (enamel and dentin). The hypothesis of this study is that the thicker the layer of lining material, the more radiopaque is its radiographic image. Another hypothesis that has been tested is that lining materials have different optical densities from those of the dental structures, irrespective of their thickness, allowing a differential diagnosis of its radiographic image.

## MATERIALS AND METHODS

To conduct this study, the materials listed in Table 1 were selected.

**Table 1:** Materials used in the study

Material	Commercial brand	Manufacturer
Calcium hydroxide cement	Hydro-C	Dentsply (Duque de Caxias, RJ, Brasil)
Glass ionomer cement	Vivaglass liner	Ivoclar / Vivadent (Schaan, Liechtenstein)

**Figure 1:**
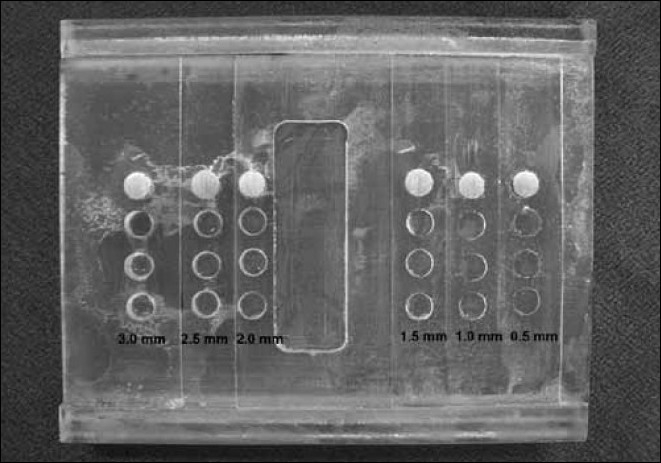
Standardizing device used to determine optical density of the tested materials

All the geometrical factors that might interfere in the formation of the radiographic image were standardized by creating a transparent acrylic matrix called the Standardizing Device, measuring 8 × 7 cm. These measurements corresponded to the width and length of an occlusal radiographic film, which was adapted to the bottom part of the device, fixed on lateral channels [Figure 1]. The upper part of the device was composed of steps that gradually increased in thickness every 0.5 mm up to the thickness of 3.0 mm. Therefore, the first step was 0.5 mm thick, the second 1.0 mm, and so on, up to the last step of 3.0 mm thickness, which was a total of six steps. On each step, three circular orifices measuring 5 mm in diameter were opened, to place the material to be radiographed, totaling to 18 orifices.

An opening was made in the center of the thickness Standardizing Device, suitable for adapting the aluminum scale (penetrometer), measuring 10 × 32 mm, and scaled in eight steps, with incremental thicknesses of 2.0, 4.0, 6.0, 8.0, 10.0, 12.0, 14.0, and 16.0 mm, according to the recommendations of Manson-Hing and Bloxom.[27] The purpose of this penetrometer was to simulate, by means of shade nuances produced after exposure and processing of the radiographs, the densities of the structures of the oral cavity in comparison to the hard and soft tissues, for laboratorial analyses of the quality of the radiographic images, to show the homogeneity of the procedures, and to detect possible variations during the procedures of the radiographic techniques and processing.

To certify that the acrylic material of the thickness Standardizing Device would not interfere with the radiographic image of the materials, an initial radiographic image was taken with an occlusal film (Insight, Kodak, New York, NY, USA) and the penetrometer only.

**Figure 2:**
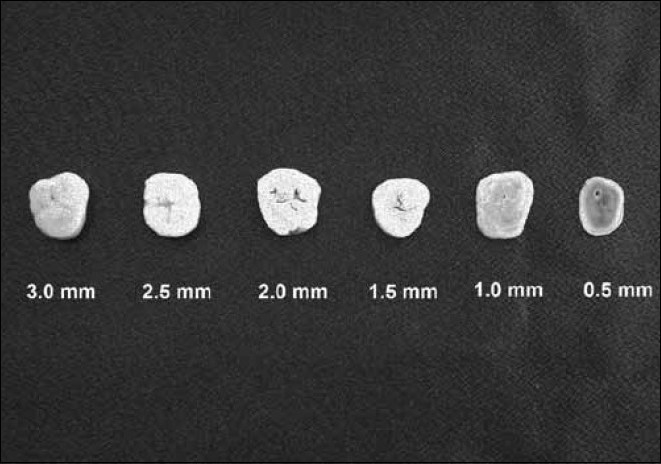
Dental cuts (cervical region - mesio-distal direction)

After receiving approval from the Research Ethics Committee of the Dentistry School of Ribeirão Preto - University of São Paulo (Process N° 2005.1.1374.58.0), the study was conducted to compare the radiopacity of the material with the dental structures (enamel and dentin). Dental cuts increasing in thickness from 0.5 to 3.0 mm were obtained from six healthy adult teeth (first molar), with one cut being taken from each tooth. These slices were acquired by sectioning the tooth in the mesio-distal direction with a diamond disk (Buehler, Lake Bluff, IL, USA) cut in a microtome (MTI Corporation, Richmond, CA, USA), in order to obtain regular dental slices with enamel and dentin, with thicknesses of 0.5, 1.0, 1.5, 2.0, 2.5, and 3.0 mm [Figure 2]. The slices were stored in artificial saliva in an oven at a temperature of 37.5°C before performing the experiment.

To regularize the two sides of these cuts and in order to assure that they presented the recommended thickness for the experiment, the dental cuts were submitted to polishing, using 800 grain water abrasive papers (Norton Abrasivos Brasil, São Paulo, SP, Brazil), in a manual polishing machine. The thickness was strictly controlled with the aid of a digital pachymeter (Digimess, São Paulo, SP, Brazil).

**Figure 3:**
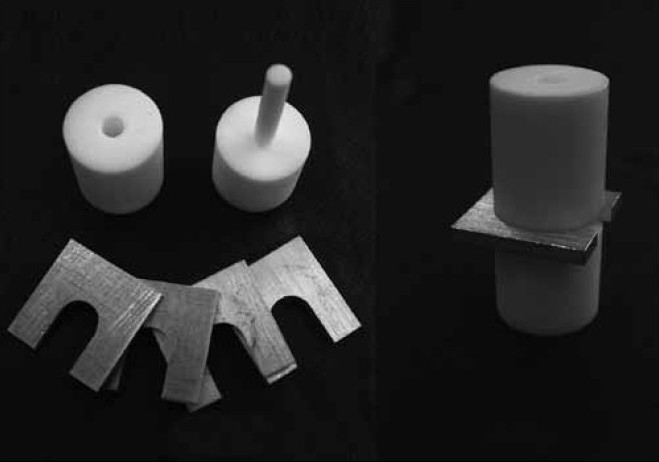
Teflon matrix used to make the test specimens

The test specimens of each material were obtained at thicknesses of 0.5, 1.0, 1.5, 2.0, 2.5, and 3.0 mm by manipulating the materials strictly in accordance with the manufacturers’ recommendations. Thus, after manipulation, the materials were immediately placed in a Teflon matrix formed by two parts fitted together: one external and the other an internal portion, in the form of a plunger, with an internal diameter of 5 mm. Accompanying the matrix, the spacers were placed according to the thickness of the test specimen that was to be obtained. These spacers were fitted into the plunger between the top and bottom portion of the matrix, so that the external part of the matrix would provide the adequate thickness of the test specimen [Figure 3]. After the material had set, the spacer was removed by lowering the matrix plunger, which released the test specimen.

**Figure 4:**
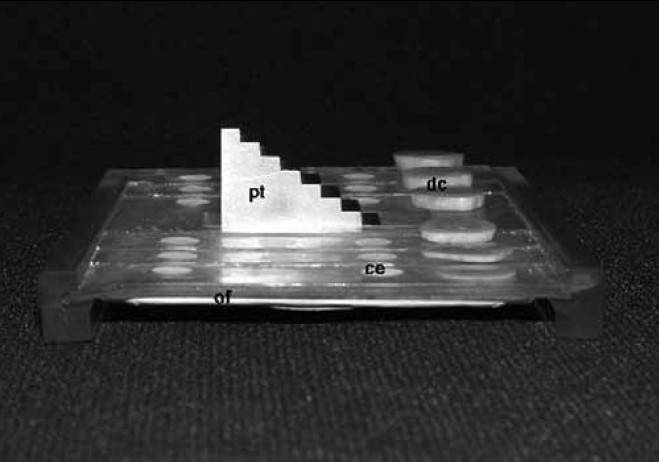
Standardizing device assembled with cements (ce), dental cuts (dt), occlusal film (of), and penetrometer (pt)

Eighteen test specimens of each studied material were made, three of each thickness. After obtaining the test specimens, they were fitted in the orifices of the matrix and radiographed. For this procedure, occlusal radiographic films (Insight, Kodak, Nova York, NY, EUA) sensitivity group ‘E’ (REF 1169143, Lot 4107790) and an X-ray appliance (Dabi Atlante, Ribeirão Preto, SP, Brazil) with a central opening, 11 mm in diameter, were used. Pilot tests were conducted to determine the exposure time of 0.5 seconds, which was defined as that which presented an optical density of 1 (+) in the radiographic image of 10 mm step wedge thickness. In addition to the materials tested, the dental disks with thicknesses according to the step height were placed next to the orifices on each step [Figure 4].

The locator cylinder was adjusted perpendicular to the Standardizing Device, at a focus-film distance of 20 cm and set for 70 kVp, with exposure time of 0.5 seconds. After taking the radiographs, the films were submitted to a manual developing process, following the manufacturer’s recommendations with regard to the time / temperature processing method.

Thirty radiographs were taken, five for each material tested. The optical density value (OD) readouts were performed with the aid of a photodensitometer (Gafchromic, Victoreen, Moedling, Austria) with the collimated light beam of 1 mm in diameter. Three OD readouts were made for each step of the penetrometer, for each sample and dental cut (enamel and dentin) per thickness.

After the readouts and after verifying the normality of the sample (Kolmogorov-Smirnov test), the means and respective standard deviations of the optical density values of each tested material were calculated, which were submitted for statistical analysis (two-way ANOVA - Bonferroni, *P* < 0.05).

## RESULTS

**Figure 5:**
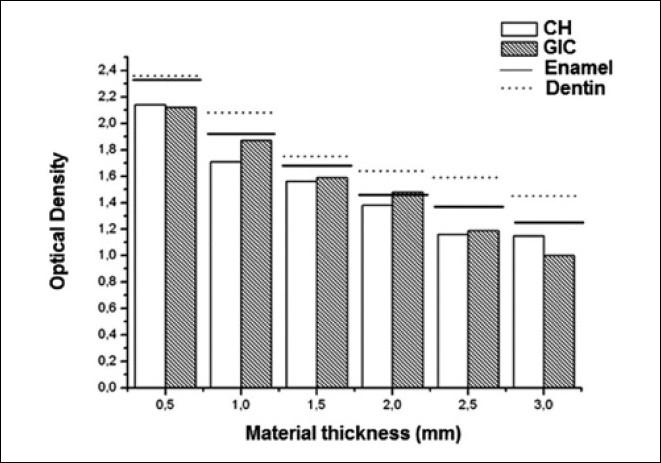
Graphic representation of the comparison of OD means of the tested materials and the dental structures

Comparison of the OD means of the materials and dental structures may be seen in Table 2 and Figure 5. Analyses of the data allowed one to verify that as the thickness increased, the OD diminished; that is, the image became gradually more radiopaque. A mean variation in OD between 1.25 and 2.33 was shown for the enamel, and from 1.45 to 2.36 for dentin. This indicated that dentin showed more radiolucence than enamel at all thicknesses; however, there was a statistically significant difference (*P* < 0.05) only from 2.0 mm.

**Table 2:** Mean optical density values (SD) for the tested materials, enamel and dentin (two-way ANOVA, Bonferroni test, *P* < 0.05)

	CH	GIC	Enamel	Dentin
0.5	2.14 (0.14)^a,A^	2.12 (0.08)^a,A^	2.33 (0.06)^b.A^	2.36 (0.11)^b.A^
1.0	1.71 (0.09)^a,B^	1.87 (0.03)^b,B^	1.92 (0.07)^c.B^	2.08 (0.05)^c.B^
1.5	1.56 (0.02)^a,C^	1.59 (0.11)^ab,B^	1.68 (0.07)^ab.C^	1.75 (0.10)^b.C^
2.0	1.38 (0.04)^a,D^	1.48 (0.04)^a,B^	1.46 (0.04)^a.D^	1.64 (0.03)^b.C^
2.5	1.16 (0.02)^a,E^	1.19 (0.02)^a,C^	1.37 (0.05)^b.DE^	1.59 (0.04)^c.C^
3.0	1.15 (0.03)^ab,E^	1.01 (0.02)^b,D^	1.25 (0.07)^a.E^	1.45 (0.08)^c.D^

Different letters, lower case in columns and capitals in lines, indicate significant difference

The mean OD of CH ranged from 1.15 to 2.14. At all thicknesses, its OD was lower than that of the dental structures, differing statistically from dentin at all thicknesses, and from enamel at the thicknesses of 0.5, 1.0, and 2.5 mm (*p* < 0.05). CIG also showed an OD lower than that of the dental structures, with the exception of thickness 2.0 mm, which presented a higher OD than the same enamel thickness, however, with no statistically significant difference (*P* > 0.05).

When comparing the materials between them, it was verified that there was a statistically significant difference (*P* < 0.05) between the OD only for the thickness of 1.0 mm. For the other thicknesses, there was no statistically significant difference (*P* > 0.05).

## DISCUSSION

Radiopacity is an important scientific property of dental materials and can be simply defined as the inverse of the optical density of a radiographic image. Optical density provides a measure of how dark the developed film can appear, as perceived by the human eye.[28] It consists of two inherent values: fog and base density. In the absence of any exposure of the undeveloped film to X-rays, a developed film will nevertheless show some absorption of light. This is called fog. Under normal conditions of measurement, the optical density of the processed emulsion of the film will be indistinguishable from the fog, due to light absorption and scattering components. However, the radiographic film for dental use is usually sold with a blue- or gray-tinted base, to improve the image visualization by the human eye and this is known as base density.[28]

The base and fog value is the inherent optical transmission density (lowest density) of a film base plus the non-image density contributed by the developed emulsion.[29] Optical density is a logarithmic measure of the ratio of transmitted to incident light through the film image, and depends not only on the inherent X-ray absorption properties of the materials, but also on the characteristics of the film, its exposure parameters, and processing conditions.[30] In this study, all these variables were standardized. Thus, any difference in optical density and hence in radiopacity was due to the different properties of materials.

The studied materials were shown to have radiopacifiers in their composition appropriate to the formation of their radiographic images.[14,15] One of the hypotheses tested showed that the thicker the layer of lining material, the more radiopaque it had in its radiographic image. The analyses of the results allowed one to agree with the hypotheses tested only for GIC. However, for CH, there was no statistically significant difference in the optical density from the thickness of 2.5 mm in comparison with the thickness of 3.0 mm (*P* > 0.05). This demonstrated a limitation in the penetration power of the X-ray into CH; that is, irrespective of the increase in thickness of the material, there was a tendency to maintain these OD values.[23,31]

Although there was no statistically significant difference (*P* > 0.05) between the OD of the lining materials at the studied thicknesses, the results indicated that visualization of the materials was possible, but it was not possible to recognize the material that was present. According to the results found, this differentiation was only possible at a thickness of 1.0 mm.

At 0.5 mm, the percentage of radiopacifier was small in comparison to the volume of the material used. The radiographic image of the material was shown to be radiolucent; that is, the amount of material present was not capable of blocking the X-rays along their trajectory, preventing sensitization of the film.[32] However, at 1.0 mm the amount of radiopacifier was sufficient to allow this sensitizing and there was differentiation in the OD of the materials, due to the presence of different metals in their compositions.[16,23] At 1.5 mm, this differentiation in OD no longer existed, because the volume of the radiopacifier agents was no longer relevant and the thickness of the material was predominant for the formation of the radiographic image.[33]

The other hypothesis tested was that the OD of lining materials differed from those of the dental structures, irrespective of their thickness. Analyses of the results did not allow one to agree with the tested hypothesis, because the radiographic images of the dental structures were confounded with the structures of the materials at some of the thicknesses, as occurred at the thicknesses of 1.5 and 2.0 mm.[9] Moreover, the dental structures were seen to have a similar OD up to a thickness of 1.5 mm. This was important for differential clinical diagnosis, because it was not possible to distinguish the three structures in the radiographic image. This phenomenon could have occurred because at a thicknesses of up to 1.0 mm, the dental structures were not sufficiently thick to prevent the passage of the X-rays, and formed a radiolucent image on the film.[33]

The OD value obtained for the enamel and dentin in this study is similar to that found by Williams and Billington,[34] who have observed a radiopacity value of 2.0 mm of aluminum for enamel and 1.0 mm for dentin. These findings are also in agreement with van Dijken et al.,[35] who has shown that the OD of dentin is approximately equivalent to that of aluminum of the same thickness and that enamel is approximately two times more radiopaque than aluminum.

With the 1.5 mm thickness, the results indicated that there was an equivalence between the OD of the dental and material structures, demonstrating that the composition of the lining materials was relevant for the formation of the radiographic image.[14,16,23] This fact became clinically relevant, because the use of these materials in the cavity to be restored was indicated for a thickness of 1.5 and 2.0 mm.[23] In the event that the remaining tooth presented enamel and dentin thicknesses similar to these, there would be no distinction in the radiographic image.

## CONCLUSIONS

Analyses of the results allowed the following conclusions to be drawn:

- The thickness of the lining material contributes to its radiopacity. However, for the CH cement, this interference is limited to 2.0 mm.
- It is not possible to differentiate the radiographic images of enamel and dentin at a thickness of up to 1.5 mm.
- The lining materials must not be used at a thickness between 1.5 and 2.0 mm in cases where there are similar thicknesses of dental structures, as it is not possible to differentiate the radiographic images.
